# Curcumin Loaded-PLGA Nanoparticles Conjugated with Tet-1 Peptide for Potential Use in Alzheimer's Disease

**DOI:** 10.1371/journal.pone.0032616

**Published:** 2012-03-05

**Authors:** Anila Mathew, Takahiro Fukuda, Yutaka Nagaoka, Takashi Hasumura, Hisao Morimoto, Yasuhiko Yoshida, Toru Maekawa, Kizhikkilot Venugopal, D. Sakthi Kumar

**Affiliations:** 1 Bio Nano Electronics Research Center, Graduate School of Interdisciplinary New Science, Toyo University, Kawagoe, Saitama, Japan; 2 Department of Respiratory Medicine, Sooriya Hospital, Chennai, India; Aristotle University of Thessaloniki, Greece

## Abstract

Alzheimer's disease is a growing concern in the modern world. As the currently available medications are not very promising, there is an increased need for the fabrication of newer drugs. Curcumin is a plant derived compound which has potential activities beneficial for the treatment of Alzheimer's disease. Anti-amyloid activity and anti-oxidant activity of curcumin is highly beneficial for the treatment of Alzheimer's disease. The insolubility of curcumin in water restricts its use to a great extend, which can be overcome by the synthesis of curcumin nanoparticles. In our work, we have successfully synthesized water-soluble PLGA coated- curcumin nanoparticles and characterized it using different techniques. As drug targeting to diseases of cerebral origin are difficult due to the stringency of blood-brain barrier, we have coupled the nanoparticle with Tet-1 peptide, which has the affinity to neurons and possess retrograde transportation properties. Our results suggest that curcumin encapsulated-PLGA nanoparticles are able to destroy amyloid aggregates, exhibit anti-oxidative property and are non-cytotoxic. The encapsulation of the curcumin in PLGA does not destroy its inherent properties and so, the PLGA-curcumin nanoparticles can be used as a drug with multiple functions in treating Alzheimer's disease proving it to be a potential therapeutic tool against this dreaded disease.

## Introduction

Alzheimer's disease (AD) is a growing concern in the modern world. It is the most common type of dementia affecting mainly the elderly population. With the better living conditions and longer life span, the number of individuals affected with AD is increasing exponentially [1], [2]. Some of the major changes occurring during AD in the brain are the accumulation of amyloid plaques, tau protein hyperphosphorylation, mitochondrial dysfunction, oxidative and inflammatory stress. The current medications do not seem to be too optimistic in halting the disease, and due to the multifactorial nature of the disease a number of factors have to be taken care of while tackling AD. As a result of this multifactorial and heterogeneous nature of the disease, compounds with multiple properties are very good candidates for treating AD [2]–[5]. Curcumin is such a versatile compound having multitude of properties. Curcumin is the major polyphenolic compound of *Curcuma longa*, a herbaceous tuberous plant endemic to South Asia. Curcumin (M.W 368.37) is chemically diferuloymethane (C_21_H_20_O_6_) and is a mixture of three major curcuminoids- curcumin, demethoxy curcumin and bis-demethoxy curcumin (Structure of curcumin: Figure: S1-A). It has a broad spectrum of biological and pharmacological activities which has been utilized since ancient times. Anti-oxidant, anti-inflammatory, anti- cancerous, anti-microbial, anti-parasitic, anti-cholesterol, anti- mutagenic properties, wound healing properties, cardiovascular, hepatic and neuronal protective activity are a few of the attributes of curcumin [6]–[8]. Curcumin can modulate multiple transcription factors, cytokines, growth factors, kinases and other enzymes. While the intravenously or intraperitoneally administered, curcumin is metabolized to tetrahydrocurcumin, hexahydrocurcumin, dihydroferulic acid and ferulic acid; the orally administered curcumin is excreted mainly through feces in unchanged form [6].

Apart from the anti-amyloid properties [8]–[11] and anti-tau hyperphosphorylation properties [12] against the two major pathological changes in AD, curcumin is also able to regulate the secondary changes that occur during the disease like oxidative stress [12], [13], inflammatory stress [11], [14] and cholesterol regulation [8], [11] which are very beneficial while considering AD therapy. Mohrko and co workers have found that similar to the amyloid binding capacity [9], [15], curcumin also shows affinity towards tau-proteins [16]. As a result of an *ab initio* computational study [17], it was reported that the unique charge and binding capabilities of curcumin even facilitates its blood-brain barrier penetration, which is very important in the case of AD. In a study conducted by Sharma and co-workers [18], it was reported that curcumin is able to improve membrane homeostasis, neuronal signaling and cognitive defects after an induced traumatic brain injury.

However, the application of curcumin is very limited due to its hydrophobic nature and non solubility in water [19]. A way to overcome this limitation is to prepare curcumin nanoparticles which are water soluble. Increasing the water solubility will also increase the biodistribution and bioavailability of curcumin, and also slow down the rapid metabolism and systemic elimination which usually occurs [7], [20]–[22]. We have utilized Poly (lactic-co-glycolic acid) (PLGA) for the synthesis of curcumin nanoparticles. PLGA is a copolymer, having excellent biocompatibility and biodegradability due to which it has been approved by the Food and Drug Administration (FDA) for use in a number of therapeutic applications [23]. PLGA is synthesized by random ring-opening copolymerization of two different monomers, the cyclic dimers of glycolic acid and lactic acid (Structure of PLGA: Figure: S1-B). It is highly biodegradable due to its ester linkage which undergoes hydrolysis in water forming its two original monomers. Glycolic acid and lactic acid are the byproducts of many of the normal metabolic pathways in the body and are metabolized and eliminated efficiently through the Kreb's cycle, which gives its biocompatible and non-toxic nature [24].

The efficiency of the medication also depends on the bio-availability at the correct location. Targeting the central nervous system has always been a challenge due to its inaccessibility. The stringent blood-brain barrier due to the presence of tight junctions within the capillary endothelium severely restricts the delivery of therapeutics to the brain [25]–[27] Tosi and co-workers had successfully synthesized and targeted a PLGA nanoparticle loaded with Loperamide and conjugated with a glycosylated heptapeptide to the central nervous system [28]. Recently, retrograde axonal transport has come up as a hopeful alternative to target cargos to the brain, bypassing the blood-brain barrier [29]. With this background, we have synthesized curcumin-PLGA nanoparticle conjugated with a targeting moiety-Tet-1 peptide. Tet-1, a 12-amino acid peptide, which has the binding characteristics of tetanus toxin was identified by Boulis and coworkers through phage-display [30]. It can interact specifically with motor neurons and is capable of retrograde delivery in the neuronal cells. The effectivity of the retrograde delivery and neuronal targeting has been well studied and established by many researchers [29], [30]. The amino acid sequence of Tet-1 peptide as identified by Liu et al. is ‘HLNILSTLWKYR’ [29]. The attachment of the Tet-1 peptide to the nanoparticle was assisted by EDC/NHS coupling reaction. EDC/NHS coupling chemistry is widely used for the attachment of proteins or DNA to nanoparticles [31], [32].

The objective of this paper is to report the conjugation of Tet-1 peptide to curcumin-PLGA nanoparticles, to study the anti-amyloid and anti-oxidative properties of the nanoparticles and to analyze the in vitro uptake of the nanoparticles.

## Methods

### 1. Synthesis of PLGA-Curcumin nanoparticles

Single emulsion –solvent evaporation method, which is the best method for encapsulating hydrophobic compounds, was used for the synthesis of PLGA-curcumin nanoparticles [7]. Briefly, 200 mg of PLGA (50∶50, MW: 7,000–17,000) was dissolved in 2 ml of ethyl acetate. 20 mg of curcumin was added to this mixture and kept for complete dissolution for half an hour with intermittent vortexing. 4 ml of PVA (5%w/v) in water was used as the surfactant and the PLGA-curcumin mixture was added drop by drop into it with intermittent vortexing at high setting. After the complete addition of the PLGA-curcumin, the mixture was sonicated at 40% amplitude for one minute to create a fine emulsion. This mixture was added to 100 ml of PVA (0.3%w/v) solution in water and rapidly stirred using a magnetic stirrer for 3 hours. PVA helps in stabilizing the emulsion and helps in forming particles of small size and uniform size distribution. The nanoparticles were collected by centrifugation at 6,000 rpm for 20 minutes, and washed thrice in Milli-Q water. The synthesized nanoparticles were freeze dried and stored at −20°C till further use. The consistencies in the synthesis parameters were checked and the above mentioned parameters were found to be the most efficient one. For analyses involving nanoparticles dispersed in water, a concentration of 10 µg/ml with respect to curcumin was used.

### 2. Characterization of the nanoparticles

#### Yield and encapsulation studies

The dry weight of the nanoparticles was measured. Yield of the nanoparticle synthesis was calculated [33] by the formula:
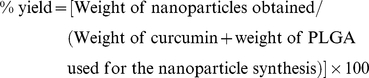
To analyze the entrapment efficiency [33] of curcumin in the nanoparticles, the amount of curcumin in the supernatant after each step of centrifugation during the synthesis of nanoparticles was quantified. A standard calibration curve was plotted using standard concentrations of curcumin dissolved in ethanol. The spectrophotometric quantification was achieved by taking the absorbance at 430 nm. The amount of encapsulated curcumin and the encapsulation efficiency was calculated using the formulae:
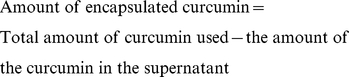


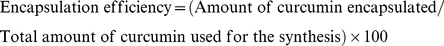



#### Physiochemical properties of the nanoparticles

Nanoparticles dispersed in Milli Q water was used for all the analyses. The size and morphology were characterized using electron microscopy. Transmission Electron Microscopy (TEM) images of the nanoparticles was taken in JEOL's JEM 2200 FS transmission electron microscope operating at 200 kV. The surface morphology was studied using Scanning Electron Microscopy (SEM) and Atomic Force Microscopy (AFM). The SEM (JEOL's JSM 7400 SEM) was operated at 5 kV. Tapping mode of the cantilever was used in the AFM analysis (Asylum Research's MFP-3D-CF AFM). The zeta potential of the particles was analyzed using the Zetasizer of Malvern Instruments. 3D TEM tomography was also performed to ascertain the encapsulation of curcumin inside the PLGA nanoparticles.

The relative change in the weight of the nanoparticle with respect to the change in temperature was studied using Thermo-gravimetric Analysis (TGA). 15–20 mg of the dry nanoparticles was placed in a Platinum pan and heated from 30 to 800°C at a heat flow rate of 10°C/min under nitrogen spurge. Attenuated Total Reflectance - Fourier Transform Infra Red Spectroscopy (ATR-FTIR) (Perkin Elmer Spectrum 100 FT-IR system) was employed to study possible interaction studies and bonding pattern between PLGA and curcumin. The Electron Spectroscopy for Chemical Analysis (ESCA/XPS) was used to check the elemental composition and chemical states of the nanoparticles. The water-dispersed samples were dropped on clean Silicon substrate and dried under vacuum and the samples were analyzed in a Kratos-Axis X-ray photoelectron spectrometer (Shimadzu, Japan) equipped with a monochromatic Al Kα X-ray source. Pass energy of 40 eV was used for the analyses. The elemental composition was calculated and curve-fitting performed using Spectral Data Processor v4.3 software.

### 3. Coupling of Tet-1 peptide and PLGA-curcumin nanoparticles

For enabling the neuronal targeting of the curcumin nanoparticles, we have tagged it with Tet-1 peptide.

Tet-1 peptide was custom synthesized from Operon Biotechnologies. Curcumin encapsulated PLGA-nanoparticles dispersed in water was used for the attachment of Tet-1 peptide. EDC-NHS coupling was used for the attachment of the Tet-1peptide to the nanoparticles. Briefly, 100 µl of PLGA-coated curcumin nanoparticles was activated using 200 µl of 400 mM 1-Ethyl-3-[3-dimethlyaminopropyl]carbodiimide hydrochloride (EDC or EDAC) and 200 µl of 100 mM N-hydroxysulfosuccinimide (NHS). The mixture was gently shaken for one hour at room temperature. To this, 20 µl of Tet-1 peptide (1 mg/ml) was added, mixed well and kept under gentle shaking for 4 hours. After the incubation period, the nanoparticles were collected by centrifugation at 6000 rpm for 15 min and washed twice with Milli-Q water. The resultant nanoparticles were suspended in nuclease free water (1 ml) and used for further analyses.

### 4. Anti-oxidant activity of the PLGA-Curcumin nanoparticles

Curcumin has strong anti-oxidant activity [12], [13]. To analyze the effect of PLGA coating on the curcumin nanoparticle and the attachment of Tet-1 on the curcumin-PLGA nanoparticle, we tested the free-radical scavenging capacity of the nanoparticles. 1,1′-diphenyl-2-picrylhydrazyl (DPPH) was used as the source of free-radicals [34]. The nanoparticles suspended in water were used for the analysis. 4 ml of the nanoparticle was mixed with methanolic solution of DPPH (100 mM) and kept in dark at room temperature for 5 hours. The DPPH scavenging activity was determined spectrophotometrically(V-650 spectrophotometer, Jasco, Japan) at 520 nm against DPPH solution as control. The samples were tested in triplicates. The anti-oxidant activity was calculated as percentage of DPPH that was decreased in comparison with the control and was calculated with the formula:
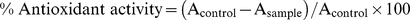



### 5. Anti-amyloid activity of PLGA-curcumin nanoparticles

The anti- amyloid activity of curcumin is a well established fact [8]–[11]. We have obtained amyloid protein (1–42 fragments) from Genscript, and dissolved it in PBS. It was left in 37°C for two weeks to initiate aggregate formation. After the 2 week incubation period, we have added PLGA-curcumin nanoparticles to it, and incubated at 37°C for different intervals of time. The samples were analyzed under SEM. The anti-amyloid activity of the Tet-1 coupled curcumin-PLGA nanoparticles were also studied similarly. The Tet-1 coupled curcumin-PLGA nanoparticles and amyloid protein aggregates were incubated at 37°C for different intervals of time and the samples were analyzed under SEM.

### 6. Cytotoxicity studies

The cytotoxicity profile of the nanoparticles was studied using two different assays- Alamar Blue and MTT assays. The cytotoxicity profile of PLGA nanoparticles (without curcumin), raw curcumin, PLGA-curcumin nanoparticles and PLGA-curcumin-Tet-1 nanoparticles were studied. Three different concentrations of each sample were tested in LAG cell line (mouse fibroblast like connective tissue). The cell lines were obtained from RIKEN Bioresource Center, Japan. The cells were grown in DMEM+10% FBS. Cells were seeded in the 96-well plates at a density of 5×10^5^cells/well and incubated at 37°C in CO_2_ incubator with 5% CO_2_ for 24 hours before the addition of the nanoparticles. After the addition of the nanoparticles, the plates were further incubated for another 24 hours before proceeding with the particular assay. Both the assays-Alamar Blue (Invitrogen) and MTT (Sigma) were performed according to the manufacturer's protocols. In Alamar Blue assay, metabolically active cells convert the non-fluorescent resazurin to fluorescent resorufin. The amount of fluorescence produced is proportional to the percentage of viable cells. The fluorescence or absorbance can be measured at 560/590 nm (Excitation/Emission) or 570 nm respectively. In MTT assay, the respiring cells (live cells) reduce the yellow tetrazolium salt to purple formazan crystals by dehydrogenase enzymes secreted by the mitochondria of metabolically active cells. Thus the amount of formazan crystals formed is proportional to the number of viable cells. The absorbance was taken at 560 nm against a background absorbance taken at 690 nm (Power scan HT Microplate Reader, Dainippon Sumitomo Pharma, Japan). Untreated cells were taken as positive controls with 100% viability and cells without adding the assay reagents were used as a blank. A negative control was maintained for validation of the results and hydrogen peroxide served as the reagent for negative control [35]. The relative cell viability was calculated using the formula:
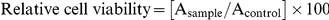



### 7. In vitro uptake study

Flow cytometry analysis was used to study the cellular uptake of the Tet-1 conjugated and unconjugated curcumin-PLGA nanoparticles. The experiments were conducted in GI-1 glioma cells. Cells were grown to attain a confluency of 10^7^cells/ml. Nanoparticles (Tet-1 attached and unattached) were added to the cells and incubated for 4 hours. After the incubation period, the cells were washed twice with 1× Phosphate buffer saline (PBS) to remove all the unbound nanoparticles. The cells were trypsinized and resuspended in 500 µl 1× PBS. Cell fixation was achieved by treating the cells with 500 µl 3% paraformaldehyde for 20 minutes. After fixation, the cells were again washed with 1× PBS and permeabilized using 500 µl ice cold methanol and incubated for 10 minutes. After adding 500 µl 1× PBS, cells were again washed and finally resuspended in 1× PBS (1 ml). To reduce cell shock, all the steps were carried out in ice. Since curcumin-PLGA nanoparticles are auto-fluorescent in FITC channel, no other cell staining technique was used.

### 8. In vitro imaging studies

The uptake of the nanoparticles were studied using confocal laser scanning microscope (CLSM) coupled into an inverted microscope (Olympus IX81, Japan) and the images were recorded using a thermoelectrically cooled CCD camera (ANDOR IQ). All the images were taken using a 100× oil immersion objective lens. GI-1 cells grown in DMEM supplemented with neurotrophic growth factors to induce differentiation were seeded on glass bottom confocal plates. After 24 hours of growth, nanoparticles were added to the cells and incubated for 4 hours at 37°C in CO_2_ incubator with 5% CO_2_ for uptake by the cells. The curcumin nanoparticles are fluorescent in the blue laser channel. To visualize the nucleus of the cells, the cells were stained with DAPI (4′, 6-diamidino-2-phenylindole) nuclear stain, which could be observed under the UV laser.

## Results

### 1. Synthesis and characterization of PLGA-Curcumin nanoparticles

**Figure 1:**
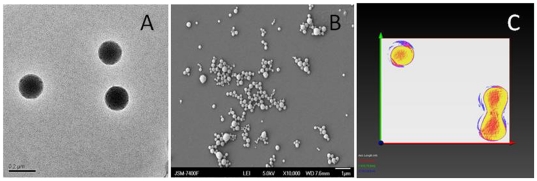
Electron microscopy studies. A- TEM image of curcumin encapsulated nanopaticle; B- SEM image of curcumin-encapsulated nanoparticle, C- 3D-TEM tomography image of curcumin encapsulated nanoparticle.

Curcumin encapsulated PLGA nanoparticles were successfully synthesized by solvent evaporation method. Consistency was checked for different batches of the nanoparticles synthesized. (We have observed that when the concentration of curcumin was increased (more than 20 mg for 200 mg PLGA) during the nanoparticle synthesis, uncoated curcumin crystals were found among the nanoparticles on examination under SEM.) The synthesized nanoparticles were completely soluble in water and showed fluorescent properties under UV light. Raw curcumin suspended in water is not soluble and do not show any fluorescence under the UV light (Figures S2 and S3). The synthesized nanoparticles were monodispersed and varied in size from 150–200 nm (Figure 1A & 1B). The PLGA coating was complete and provided a smooth surface to the nanoparticles. The zeta potential of the nanoparticles was in the range −30 to −20 mV. The 3D TEM tomography confirmed the encapsulation of the curcumin inside the nanoparticles. A computer generated slice-image obtained after compiling all the images taken at different angles show that curcumin is encapsulated inside the nanoparticle, which is recognized from the difference in the density of the two materials as observed in the image obtained (Figure 1C).

**Figure 2:**
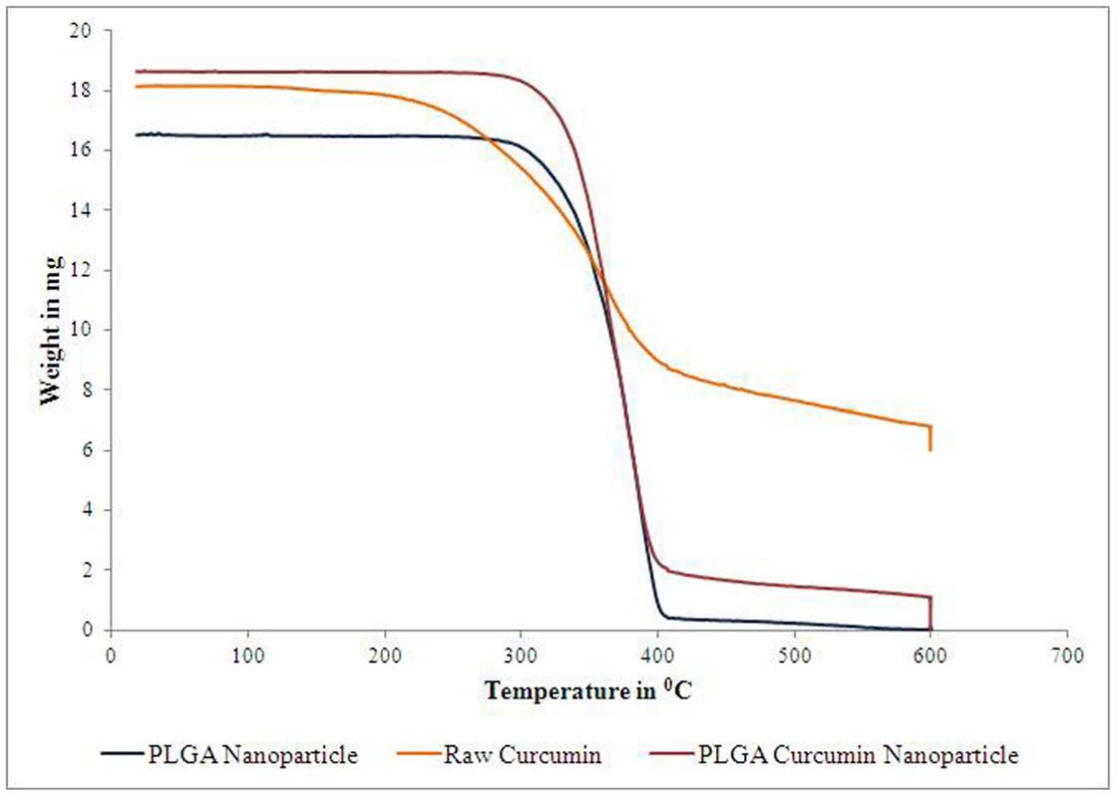
TGA Analysis of PLGA nanoparticle, raw curcumin and PLGA-curcumin nanoparticle. Curcumin undergoes a gradual thermal decomposition when compared to the rapid decomposition in PLGA and PLGA-curcumin nanoparticles.

Thermogravimetric analyses of PLGA nanoparticles, raw curcumin and curcumin-encapsulated PLGA nanoparticles shows that PLGA-curcumin nanoparticle follows the weight-loss pattern of PLGA nanoparticles. The weight-loss in raw curcumin occurs around 200°C, while that of the PLGA nanoparticle and PLGA-curcumin nanoparticle occurs around after 300°C. The weight loss in curcumin follows a gradual decrease while that of PLGA and PLGA-curcumin nanoparticles occur more rapidly. The raw curcumin and the curcumin loaded PLGA nanoparticles left some residual matter after the end of thermal decomposition, while the PLGA burnt completely without leaving behind any residual matters (Figure 2). The result of the ATR-FTIR analyses show that there is no chemical interaction taking place between PLGA and curcumin (data not shown). XPS analysis helped to provide a more qualitative and definitive verification about the composition of the nanoparticles. It also gave a definitive proof regarding the attachment of the Tet-1 peptide on to the surface of the nanoparticle, which will be discussed in detail in a later section.

### 2. Coupling of Tet1 peptide and PLGA-Curcumin nanoparticles

**Figure 3:**
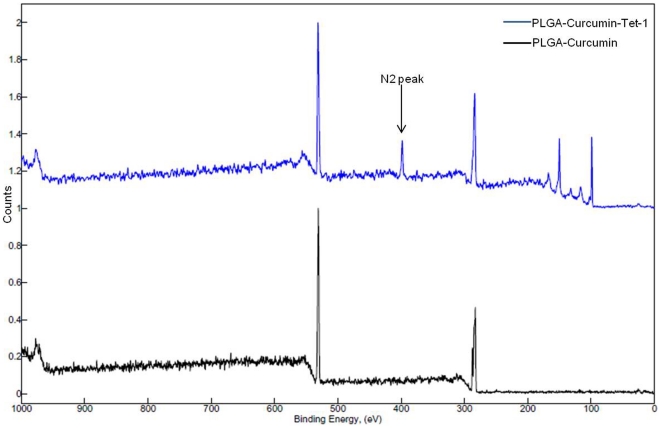
XPS spectra of nanoparticles. (A)- XPS spectra of PLGA-curcumin nanoparticles. (B)- XPS spectra of PLGA-Curcumin nanoparticle attached to Tet-1 peptide. The N2 peak which identifies the presence of Tet-1 peptide can be clearly found in the spectra. The peak at 531 eV represents Oxygen, 285 eV- Carbon and 398 eV- Nitrogen. (The peaks at 150 and 100 correspond to that of Si of the Silicon substrate- Figure S4).

Tet-1 peptide was successfully attached to the PLGA-Curcumin nanoparticles by EDC-NHS coupling reaction. The attachment of Tet-1 was confirmed by N_2_ peak in XPS (ESCA) (Figure 3). As XPS is one of the most versatile tools in identifying the chemical species on a surface, we have used it for the confirmation of Tet-1 attachment to the nanoparticles. The wide spectrum obtained shows the peaks corresponding to Carbon (284 eV), Oxygen (532 eV) and most importantly, Nitrogen (398 eV). Nitrogen peak is absent in PLGA-curcumin nanoparticle. Nitrogen is a component of the peptide (Tet-1) and the amide bond formed between the nanoparticle and peptide by NHS activation.

### 3. Anti-oxidant activity of the nanoparticles

**Figure 4:**
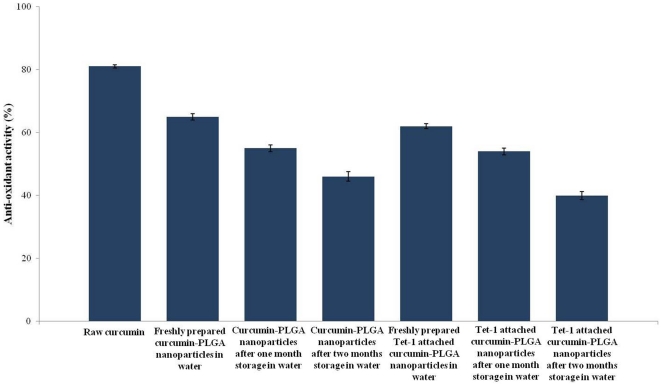
The anti-oxidant activity of curcumin was analyzed by its activity in reducing DPPH. The percentage of antioxidant activity is expressed as percentage (Y axis).

The DPPH assay was used to study the free-radical scavenging capacity of curcumin encapsulated PLGA nanoparticle in comparison with raw curcumin. The results are plotted in the graph (Figure 4).

Raw curcumin showed more than 80% free-radical scavenging activity. Freshly prepared curcumin-PLGA nanoparticles had nearly 60% free-radical scavenging activity, and the anti-oxidant activity was decreased with the long term storage of the curcumin-PLGA nanoparticles. Attachment of Tet-1 to the curcumin-PLGA nanoparticles did not produce any change in its anti-oxidant activity. Comparable anti-oxidant activity was exhibited by Tet-1 attached and Tet-1 unattached curcumin-PLGA nanoparticles. A decrease in the anti-oxidant activity was exhibited by Tet-1 attached nanoparticles on long term storage, similar to that exhibited by the curcumin-PLGA nanoparticles (without Tet-1). PLGA nanoparticles (without curcumin) did not show any free-radical scavenging activity. With lesser reaction time (30 minutes-1 hour), there was a decrease in overall free-radical scavenging activity. This is because; the encapsulated curcumin requires time to leach out into the DPPH solution for the scavenging activity to take place. The point to be noted is that the encapsulation with PLGA does not destroy the anti-oxidant activity of curcumin.

### 4. Anti-Amyloid activity of PLGA-curcumin nanoparticles

**Figure 5:**
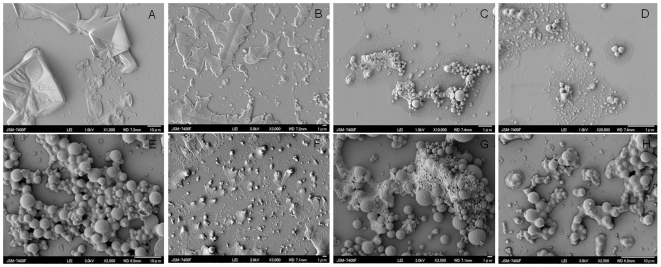
Anti-amyloid activity of PLGA-curcumin nanoparticles. A- Amyloid protein (Aβ) aggregates, B- Aβ and PLGA-curcumin nanoparticles after 12 hour co-incubation, C- Aβ and PLGA-curcumin nanoparticles after 24 hour co-incubation, D- Aβ and PLGA-curcumin nanoparticles after 48 hour co-incubation, E- Tet-1 conjugated PLGA-curcumin nanoparticles, F- Aβ and Tet-1 attached PLGA-curcumin nanoparticles after 12 hour co-incubation, G- Aβ and Tet-1 attached PLGA-curcumin nanoparticles after 24 hour co-incubation, H- Aβ and Tet-1 attached PLGA-curcumin nanoparticles after 48 hour co-incubation. It is observed that the nanoparticles are able to attach with the amyloid aggregates and help in disaggregation of the amyloid protein aggregates.

Anti-amyloid activity of curcumin is an interesting property studied by many researchers. Although the exact mechanism of curcumin activity is not known, it is an established fact that curcumin stops the aggregation of amyloid fibrils and help in the disaggregation of the existing plaques. We have obtained similar results (Figure 5) when the PLGA- curcumin nanoparticles were treated with amyloid protein aggregates. Amyloid aggregates were formed by prolonged incubation at 37°C (Figure 5A) and treated with the PLGA-curcumin nanoparticles to study its anti-amyloid activity. We have observed that in due course of time, PLGA-curcumin nanoparticles are able to slowly disaggregate the amyloid proteins. It was observed that PLGA-curcumin nanoparticles were able to attach to the amyloid aggregate surface and decrease the size of the aggregates within 12 hours of co-incubation (Figure 5B). After 24 hours of incubation, significant break down in the amyloid aggregates was observed (Figure 5C) and by 48 hours of co-incubation, the aggregates are broken down into considerably smaller plaques (Figure 5D). We have also found that Tet-1 attachment does not alter the anti-amyloid activity of curcumin. Morphology of the Tet-1 attached nanoparticles and unattached nanoparticles are similar (figure 5E and figure 1B) Tet-1 attached curcumin-PLGA nanoparticles also exhibited anti-amyloid activity similar to that of curcumin-PLGA nanoparticle (without Tet-1) (figure 5F–H). It was observed that the anti-amyloid activity of Tet-1 attached nanoparticles was slower when compared to the Tet-1 unattached nanoparticles. By 48 hours, complete breakdown of amyloid aggregates were observed on treating with curcumin-PLGA nanoparticles, while after the same duration although Tet-1 coupled nanoparticles were able to disaggregate amyloid proteins, complete breakdown took another 24 hours (total of 72 hours). However, it has to be noted that anti-amyloid activity remains unaltered after the attachment of Tet-1 to the curcumin-PLGA nanoparticle. No anti-amyloid effect was observed when the amyloid protein aggregates were treated with PLGA nanoparticles (devoid of curcumin).

### 5. Cytotoxicity studies

**Figure 6:**
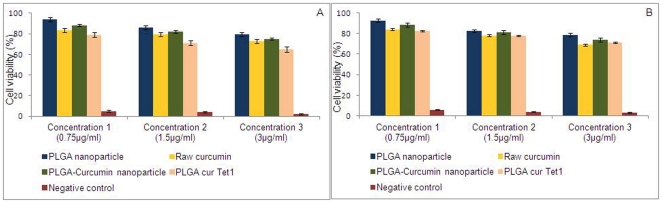
Cytotoxicity analyses results by Alamar Blue assay (A) and MTT assay (B) after 24 hour incubation with the nanoparticle. Three different concentrations of the test samples were added to the cells and incubated for 24 hours before adding the respective assay reagents. We have observed that the nanoparticles were highly biocompatible.

Cytotoxicity profile of the nanoparticles was studied using Alamar Blue assay and MTT Assay. As cancer cells are sensitive towards curcumin, we chose to use LAG, a normal mouse fibroblast like connective tissue for the cytotoxicity analyses. Three different concentrations of each of the test samples (PLGA nanoparticle, raw curcumin, PLGA-curcumin nanoparticle, PLGA-curcumin-Tet-1 nanoparticle, negative control) were used for the studies. The respective assay reagents were added after incubating the cells and the nanoparticles for 24 hours. The fluorescence and absorbance readings were taken and the result calculated. From the results obtained, it is concluded that PLGA-curcumin nanoparticles do not possess any significant cytotoxic effect on normal cells (Figure 6). The cells treated with the highest concentration of nanoparticles also showed a viability of more than 70%. Similarly, there was no significant difference in the cytotoxicity after the attachment of the nanoparticle with Tet-1 peptide. Maintaining a negative control helped in verifying the accuracy of the assays. It can be concluded that the nanoparticles are highly biocompatible and do not possess any significant toxicity in vitro.

### 6. In vitro uptake study

**Figure 7:**
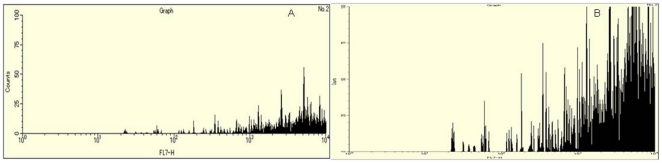
Flow cytometry analysis of the uptake of the targeted and non-targeted nanoparticles based on the fluorescent intensity of the nanoparticle uptake in GI-1 glioma cells. A- uptake of curcumin-PLGA nanoparticles; B- uptake of Tet-1 conjugated curcumin-PLGA nanoparticles. Enhanced uptake of Tet-1 attached nanoparticles were observed when compared to the curcumin-PLGA nanoparticles (without Tet-1).

Flow cytometry experiments helped in understanding the in vitro uptake of the targeted and non-targeted nanoparticles inside the cells. GI-1 glioma cells were used for the study. The uptake study was based on the fluorescence intensity of the curcumin nanoaprticles, which are fluorescent in the FITC channel. It was observed that Tet-1 targeting increased the neuronal uptake of the curcumin-PLGA nanoparticles compared to the non-targetd curcumin-PLGA nanoparticles. Figure 7 illustrates the results obtained after the flow cytometry analysis. It can be very clearely observed that the uptake of the nanoparticle is increased by multiple folds when it is targetd with Tet-1 peptide (figure 7B) compared to the non-targeted nanoparticle (figure 7A) as per the fluorescence data obtained. This proves that Tet-1 targeting can be used for drug delivery to neurons with increased efficiency.

### 7. In vitro imaging studies

**Figure 8:**
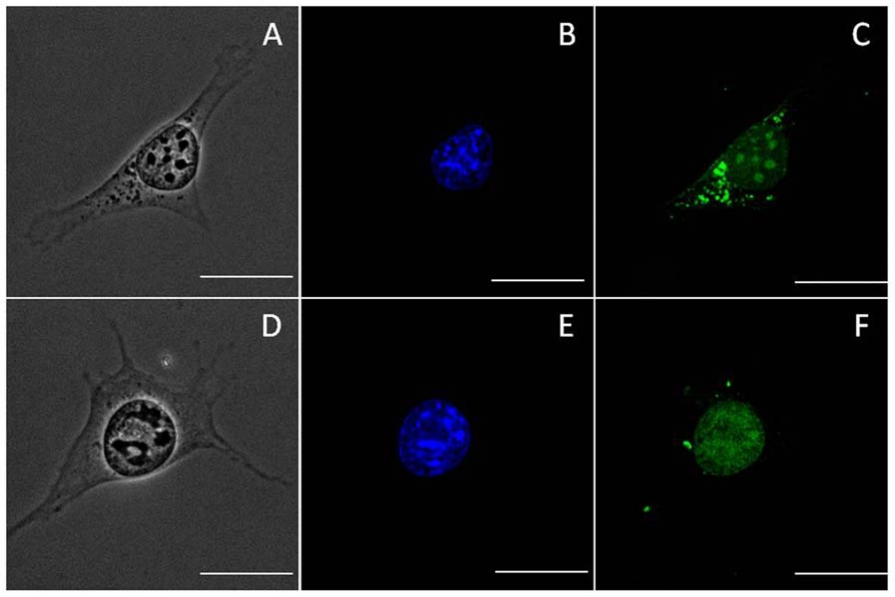
Confocal imaging of nanoparticle uptake by GI-1 cells. A, B, C- PLGA-curcumin nanoparticles (without Tet-1 peptide) are easily taken up by the cell and are found distributed all though the cell cytoplasm. D, E, F- Nanoparticles targeted with Tet-1 peptide show greater affinity towards the cell soma and nucleus. A& D- Bright-field image, B& E- stained with DAPI nuclear stain observed in blue, C&F- autofluorescent PLGA-curcumin nanoparticles are observed in green. The scale bars on all the images correspond to 20 µm.

In vitro imaging studies is an important tool in studying the targeting properties of nanoparticles. Since our curcumin nanoparticles are auto-fluorescent, it provides a greater opportunity to study its movement in vitro. Curcumin nanoparticles are visible under the blue laser in the Confocal Laser Scanning Microscope (CLSM). We have conducted the cell imaging studies in GI-1 glioma cells as a model for neuronal cells. GI-1 cells were grown in DMEM supplemented with neurotropic growth factors to reduce division and facilitate differentiation to a small extend. After incubation with the nanoparticles, the cells were washed with PBS and counter stained with DAPI nuclear stain and viewed under the CLSM. The nanoparticles are taken up inside the cells very quickly and get distributed almost equally inside the cells (Figure 8: A–C). After the addition of the Tet-1 targeted nanoparticles, a greater concentration of nanoparticles is observed around the cell soma and nucleus (Figure 8: D–F) when compared to the non-targeted nanoparticles, confirming retrograde transport of the nanoparticles.

## Discussion

### (i) Physiochemical properties of the nanoparticles

Although curcumin is a promising compound with numerous beneficial properties, its utility is greatly restricted by its insolubility in water. Water solubility is one of the important requirements for an ideal drug. We have achieved water solubility by preparing nanoparticle by encapsulating curcumin in PLGA. Apart from the complete solubility in water, these particles also exhibit fluorescent properties. The nanoparticles are also monodispersed in water which also adds to its advantage. According to Lockman et al., [36], anionic charge on the nanoparticle supports entry through the blood-brain barrier when compared to the cationic nanoparticles. PLGA-curcumin nanoparticles we have synthesized are slightly anionic. The non-reactivity between PLGA and curcumin helps in conserving the natural properties of both of these compounds. PLGA nanoparticles are taken up by the cells mainly through the clathrin-mediated endocytosis [37]. Cell viability studies prove that curcumin-PLGA and Curcumin-PLGA-Tet-1 nanoparticles are not cytotoxic. Three different Phase I clinical trials have proven that curcumin is safe at high doses (12 g/day) [19].

### (ii) Anti-oxidant and anti- amyloid properties of the nanoparticles

The anti-oxidant property of curcumin is one of the most important features which make its therapeutic value very high. In AD, formation of amyloid plaques results in severe oxidative stress in the neurons. The severe oxidative stress is also a reason for the neuronal loss which occurs during AD. The PLGA coated curcumin nanoparticles conserve the anti-oxidant property and is able to scavenge the free-radical source- DPPH on reacting with it. Although the nanoparticles are not as potent as raw curcumin, it does exhibit comparable reactivity. Attachment of Tet-1peptide to the nanoparticles did not affect its antioxidant activity.

We were able to reproduce the amyloid binding activity shown by raw curcumin in our PLGA coated curcumin nanoparticles also. The curcumin nanoparticles were able to stop the aggregation of the amyloid plaques and disrupt the aggregates which were already formed. Earlier it was reported by Garcia-Alloza and coworkers [10] that in *in vivo* mice models, curcumin was able to bind to amyloid plaques and dramatically clear them. They have also reported that curcumin was able to reduce the appearance of new plaques in these transgenic mice. They suggest that apart from microglial activation, some other indirect contribution is also provided by curcumin in decreasing the amyloid burden. Similar results were published earlier by Yang et al., [9] and Ono et al., [38]. Recently, many reports have come up with the use of curcumin nanoformulations for the treatment of AD. All of these reports agree on the anti-amyloid and anti-oxidant activity of curcumin which inhibits the fibrillar and oligomeric formations of amyloid aggregates [39]. Mourtas and coworkers have reported the importance of structural planarity of curcumin for anti-amyloid activity [40]. Apart from these studies, it has also been reported that curcumin can decrease the amyloid levels by modulating the secretory and endocytic pathways of the amyloid-precursor protein [41].

### (iii) Targeting facilitated by attachment of Tet-1 peptide to the nanoparticles

The delivery of the drug to the right target is as important as its activity. Targeting nanoparticles to treat diseases of cerebral origin are very difficult due to the presence of the blood-brain barrier. Blood-brain barrier restricts the entry of almost all particles. Due to the unique properties of curcumin, it is able to cross the blood-brain barrier [17]. The targeting of the nanoparticles with the Tet-1 peptide enhances the uptake by the neuronal cells. Moreover, there is a possibility of targeting Tet-1 conjugated nanoparticles to the central nervous system by nasal delivery. Nasal delivery of drugs is an alternative to invasive methods of drug administration. Hanson and Frey [42] have reported the successful intranasal delivery of neuroprotective peptide- NAP, in a transgenic mouse model of AD. In another study by Gao and co-workers [25], lectin-conjugated PEG-PLA nanoparticles have been utilized for cerebral delivery by intranasal administration. Similar targeting strategy has been reported by Vergoni et al., [26] in rats. They have used a glycosylated heptapeptide attached to PLGA nanoparticles for targeting central nervous system. Recently, Li and coworkers [43] have reported the successful targeting of brain with PEG-PLGA nanoparticles modified with a 12-mer phage-displayed peptide (Pep TGN). They have conducted in vivo study in mice and have found the movement of the nanoparticles across the blood-brain barrier.

### Conclusion

Treatment of Alzheimer's disease needs to take care of the multifactorial nature of the disease and the use of multi-functional drugs has been identified as the need-of the hour. Apart from the amyloid pathology, oxidative stress, inflammatory stress, tau protein hyperphosphorylation and cholesterol homeostasis plays important role in the development of the disease. Curcumin with its multiple properties can be a powerful drug in treating AD. The results of our *in vitro* study suggest that Tet-1 targeted PLGA coated curcumin nanoparticles can be potential tool in treating AD with respect to its anti-amyloid property and anti-oxidant property, which we have reported here. Tagging the PLGA- curcumin nanoparticle with Tet-1 neuropeptide greatly increases its *in vitro* neuronal targeting efficiency. Although further conclusions can only be drawn only after a thorough *in vivo* study, the results of this preliminary study suggest the potential role curcumin can play in treating AD and the effect of Tet-1 peptide in neuronal targeting of the nanoparticles. In addition to the biocompatible nature of curcumin, the long history of its therapeutic use and low cost of the material also adds on to the advantages and makes it a promising candidate drug for treating AD.

## Supporting Information

Figure S1 **Structure of curcumin (A) and PLGA (B).**
(TIF)

Figure S2 **Solubility of curcumin under visible light: A- Curcumin dissolved in water, B- Curcumin-PLGA nanoparticles in water, C- Raw curcumin dissolved in acetone.**
(TIF)

Figure S3 **Solubility of curcumin under UV: D- Curcumin dissolved in water, E- Curcumin-PLGA nanoparticles in water, F- Raw curcumin dissolved in acetone.**
(TIF)

Figure S4 **XPS spectra of Si substrate used for the analysis of the samples.** The peak at 531 represent Oxygen (1 s) peak, 285 represent Carbon peak (1 s) and the peaks at 151 and 99 represent the 2 s and 2p peaks of Silicon respectively.
(TIF)
