# Supplementary figures and images for: Curcumin Loaded-PLGA Nanoparticles Conjugated with Tet-1 Peptide for Potential Use in Alzheimer's Disease
Source: PLoS One. 2012 Mar 5;7(3):e32616. doi: 10.1371/journal.pone.0032616 (PMC3293842; doi:10.1371/journal.pone.0032616)

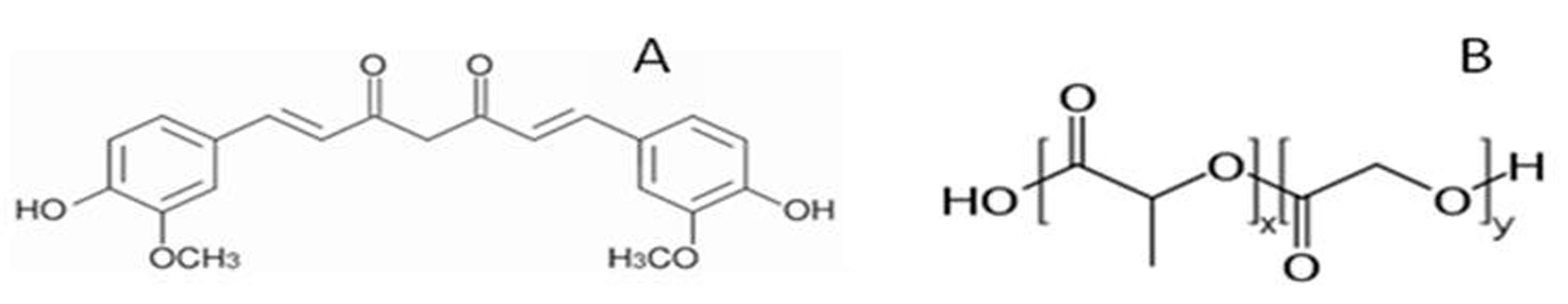

Supplement: Figure S1 — Structure of curcumin (A) and PLGA (B). (TIF) [file pone.0032616.s001.tif]

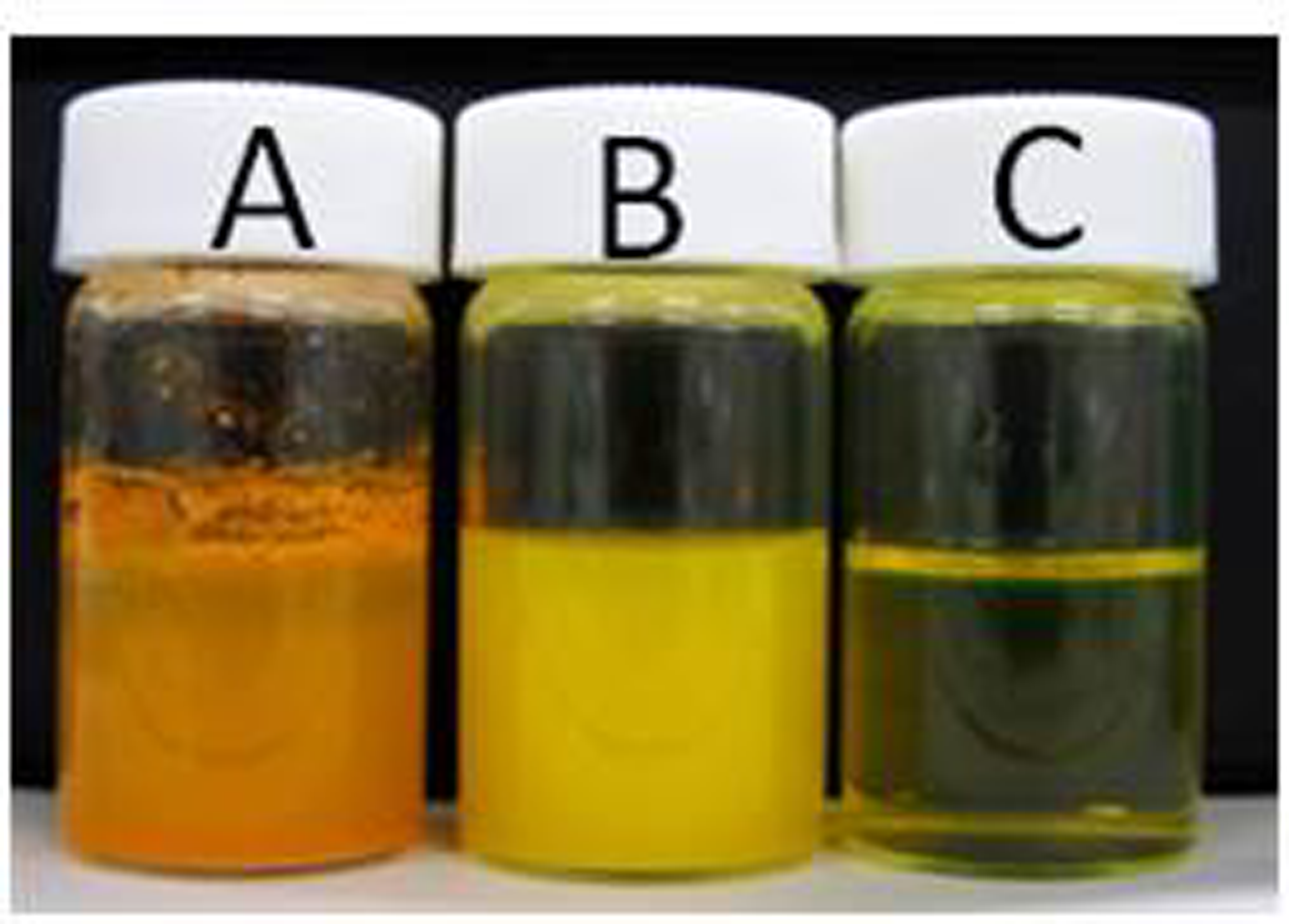

Supplement: Figure S2 — Solubility of curcumin under visible light: A- Curcumin dissolved in water, B- Curcumin-PLGA nanoparticles in water, C- Raw curcumin dissolved in acetone. (TIF) [file pone.0032616.s002.tif]

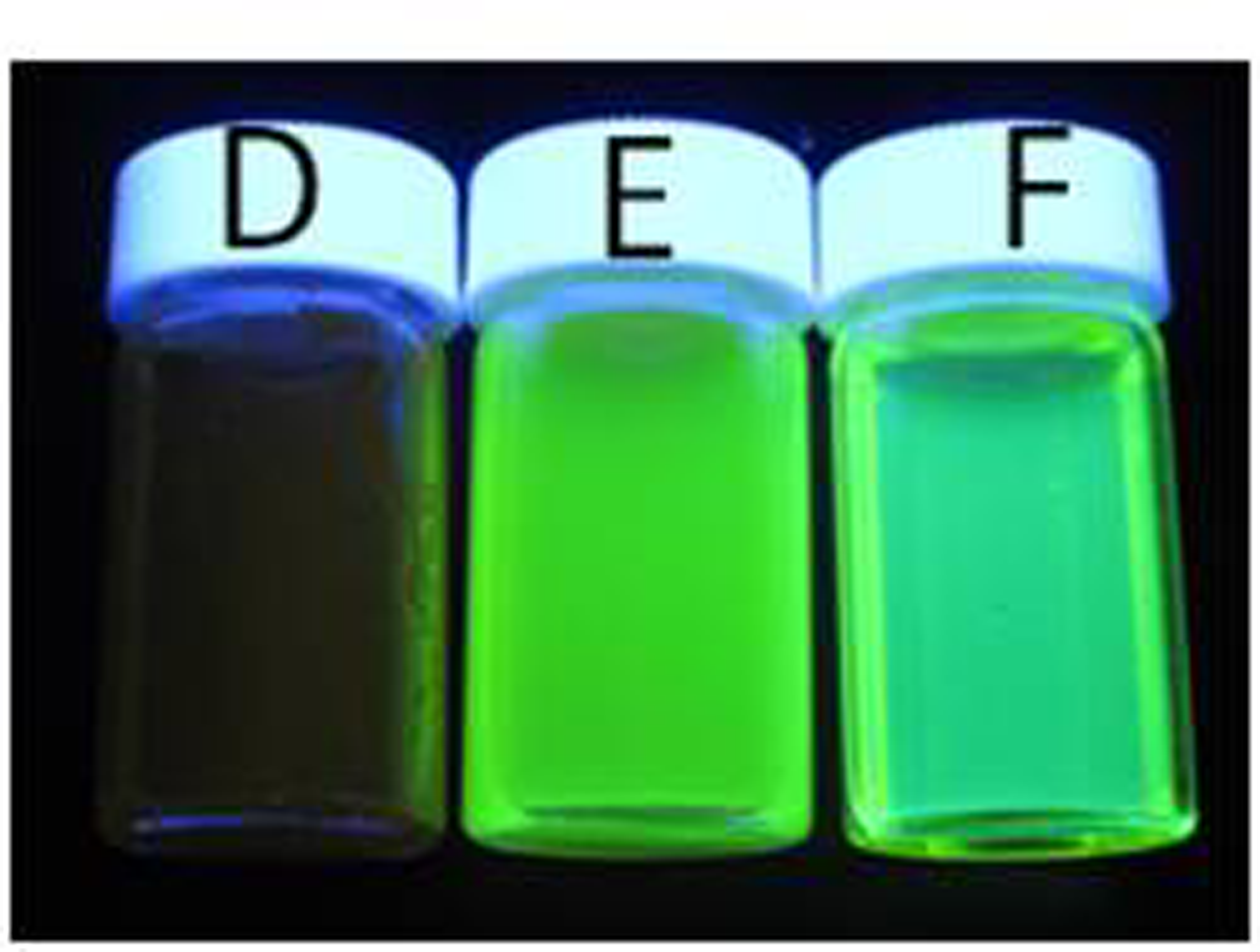

Supplement: Figure S3 — Solubility of curcumin under UV: D- Curcumin dissolved in water, E- Curcumin-PLGA nanoparticles in water, F- Raw curcumin dissolved in acetone. (TIF) [file pone.0032616.s003.tif]

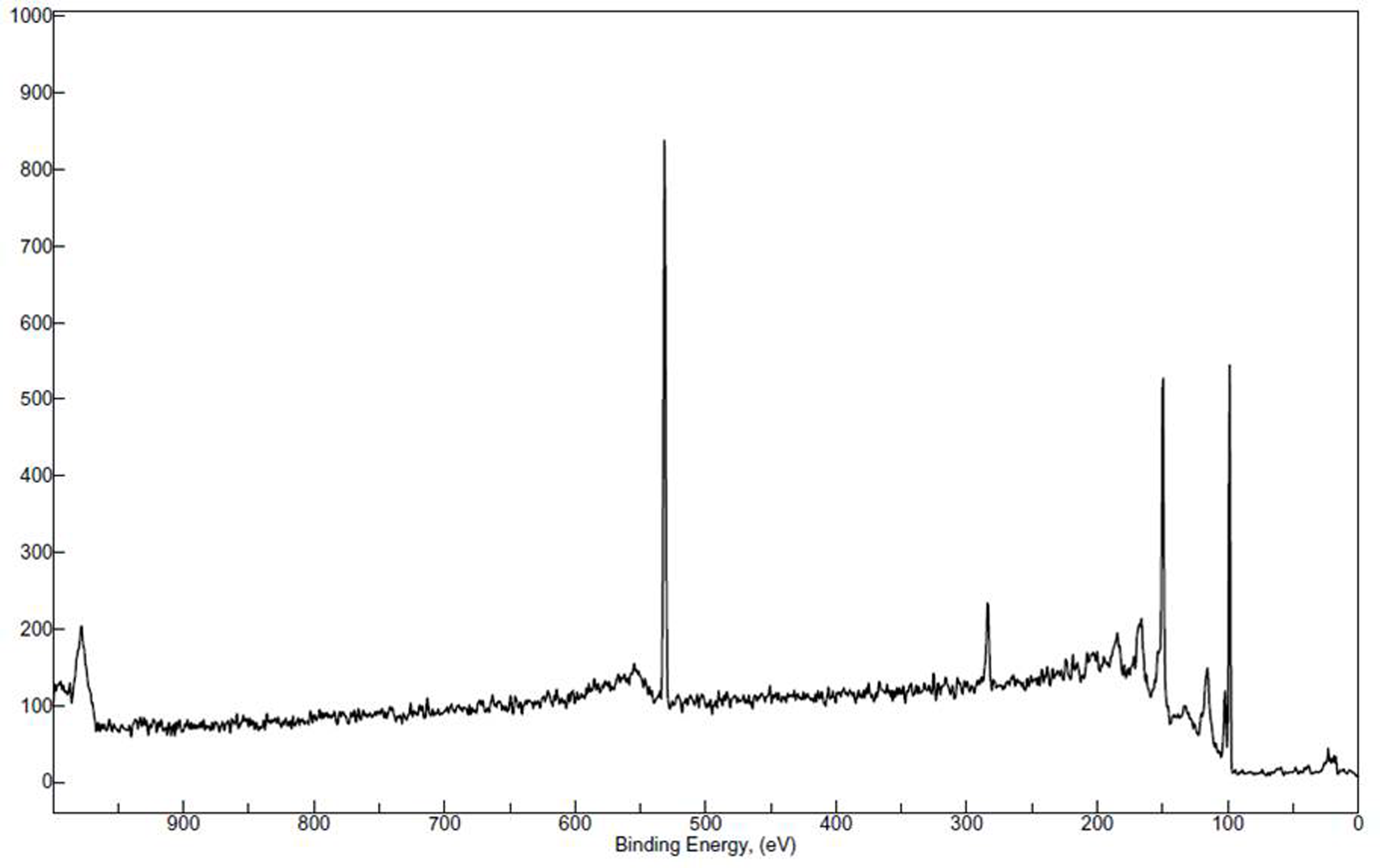

Supplement: Figure S4 — XPS spectra of Si substrate used for the analysis of the samples. The peak at 531 represent Oxygen (1 s) peak, 285 represent Carbon peak (1 s) and the peaks at 151 and 99 represent the 2 s and 2p peaks of Silicon respectively. (TIF) [file pone.0032616.s004.tif]
